# Association Study of 167 Candidate Genes for Schizophrenia Selected by a Multi-Domain Evidence-Based Prioritization Algorithm and Neurodevelopmental Hypothesis

**DOI:** 10.1371/journal.pone.0067776

**Published:** 2013-07-29

**Authors:** Zhongming Zhao, Bradley T. Webb, Peilin Jia, T. Bernard Bigdeli, Brion S. Maher, Edwin van den Oord, Sarah E. Bergen, Richard L. Amdur, Francis A. O'Neill, Dermot Walsh, Dawn L. Thiselton, Xiangning Chen, Carlos N. Pato, The International Schizophrenia Consortium, Brien P. Riley, Kenneth S. Kendler, Ayman H. Fanous

**Affiliations:** 1 Department of Biomedical Informatics, Vanderbilt University School of Medicine, Nashville, Tennessee, United States of America; 2 Department of Psychiatry, Vanderbilt University School of Medicine, Nashville, Tennessee, United States of America; 3 Virginia Institute for Psychiatric and Behavioral Genetics, Virginia Commonwealth University, Richmond, Virginia, United States of America; 4 Center for Biomarker Research and Personalized Medicine, Virginia Commonwealth University, Richmond, Virginia, United States of America; 5 Department of Mental Health, Johns Hopkins Bloomberg School of Public Health, Baltimore, Maryland, United States of America; 6 Psychiatric and Neurodevelopmental Genetics Unit, Center for Human Genetics Research, Massachusetts General Hospital, Boston, Massachusetts, United States of America; 7 Stanley Center for Psychiatric Research, Broad Institute of MIT and Harvard, Cambridge, Massachusetts, United States of America; 8 Washington VA Medical Center, Washington, DC, United States of America; 9 Department of Psychiatry, Queens University, Belfast, United Kingdom; 10 The Health Research Board, Dublin, Ireland; 11 Department of Psychiatry, Virginia Commonwealth University, Richmond, Virginia, United States of America; 12 Department of Human and Molecular Genetics, Virginia Commonwealth University, Richmond, Virginia, United States of America; 13 Department of Psychiatry, Georgetown University School of Medicine, Washington, DC, United States of America; 14 Department of Psychiatry, Keck School of Medicine of the University of Southern California, Los Angeles, California, United States of America; Yale University, United States of America

## Abstract

Integrating evidence from multiple domains is useful in prioritizing disease candidate genes for subsequent testing. We ranked all known human genes (n = 3819) under linkage peaks in the Irish Study of High-Density Schizophrenia Families using three different evidence domains: 1) a meta-analysis of microarray gene expression results using the Stanley Brain collection, 2) a schizophrenia protein-protein interaction network, and 3) a systematic literature search. Each gene was assigned a domain-specific p-value and ranked after evaluating the evidence within each domain. For comparison to this ranking process, a large-scale candidate gene hypothesis was also tested by including genes with Gene Ontology terms related to neurodevelopment. Subsequently, genotypes of 3725 SNPs in 167 genes from a custom Illumina iSelect array were used to evaluate the top ranked vs. hypothesis selected genes. Seventy-three genes were both highly ranked and involved in neurodevelopment (category 1) while 42 and 52 genes were exclusive to neurodevelopment (category 2) or highly ranked (category 3), respectively. The most significant associations were observed in genes *PRKG1*, *PRKCE*, and *CNTN4* but no individual SNPs were significant after correction for multiple testing. Comparison of the approaches showed an excess of significant tests using the hypothesis-driven neurodevelopment category. Random selection of similar sized genes from two independent genome-wide association studies (GWAS) of schizophrenia showed the excess was unlikely by chance. In a further meta-analysis of three GWAS datasets, four candidate SNPs reached nominal significance. Although gene ranking using integrated sources of prior information did not enrich for significant results in the current experiment, gene selection using an *a priori* hypothesis (neurodevelopment) was superior to random selection. As such, further development of gene ranking strategies using more carefully selected sources of information is warranted.

## Introduction

A wealth of information relevant to the genetics of complex disorders is available via a wide variety of platforms such as gene expression, protein-protein interactions (PPIs), biological pathways, and Gene Ontology (GO). It was hoped that the advent of large scale genome-wide association studies (GWAS) would eliminate the need to utilize this data as a means to uncover susceptibility loci. However, psychiatric GWAS have shown that there are likely many loci of small effect and few results are significant after corrections for multiple testing [1], [2], [3]. Furthermore, the loci that do survive only account for modest proportions of heritability. Therefore, novel methods are still needed to identify additional causative loci. The use of multiple, existing sources of information could increase statistical power to detect susceptibility genes and minimize the risk of pursuing false positives in follow up investigations. However, due to the large amount of information plus heterogeneity among data sources, the task of combining such information in an optimal way is complex and difficult, either intuitively or manually.

Schizophrenia is a disorder that is particularly suitable to this type of approach. While other complex disorders and traits such as type 2 diabetes and height have been gathering a rapidly growing list of replicated and validated susceptibility loci, several features of schizophrenia will arguably make such success less likely. Although its heritability is higher than many complex disorders such as type 2 diabetes, its prevalence is lower. This makes very large studies with tens or hundreds of thousands of participants much more challenging (albeit necessary in order to detect an effect). There is also phenotypic and diagnostic heterogeneity which is arguably less present in other complex disorders and which may reflect genetic heterogeneity as well. Moreover, for schizophrenia, there is increasing evidence suggesting a complex genetic architecture comprising a mixture of rare highly-penetrant mutations such as large deletions in gene *NRXN1* [4] as well as common single nucleotide polymorphisms (SNPs) [5]. Furthermore, well developed animal models or the availability of patient tissue are very limited. However, there are multiple schizophrenia GWAS available now, which can be used to evaluate hypotheses or ranking procedures.

We have previously developed a procedure for gene ranking based on *a priori* evidence and the results from a small validation study were encouraging [6]. Here, we reported a modified ranking procedure for complex diseases such as schizophrenia, applied it to all genes residing in regions of linkage in the Irish Study of High Density Schizophrenia Families (ISHDSF) sample, and performed a larger evaluation of the method. To evaluate the utility of this approach, we compared it with a gene selection approach based on the well-established neurodevelopmental etiological hypothesis of schizophrenia [7].

## Materials and Methods

### Ethics statement

This research was approved by the Institutional Review Boards of Virginia Commonwealth University School of Medicine and the Washington VA Medical Center. All subjects gave verbal assent to participate in research, as this represented the ethical standard in Ireland at the time these data were collected. This strategy was specifically approved by the Health Research Board, Dublin. Permission was received to use the data in this study, and the data we de-identified prior to analysis.

### Subjects and phenotypes

The Irish Study of High Density Schizophrenia Families (ISHDSF) sample consists of 265 high-density schizophrenia families with 1408 individuals available for genotyping [8]. All participating individuals gave appropriate informed consent to the study. The sample was divided into 4 concentric diagnostic categories for analysis purposes, ranging from core schizophrenia (D2, 625 affected individuals), through narrow spectrum (‘intermediate phenotype’ D5, 804 affected individuals), broad (D8, 888 affected individuals) and very broad spectrum disease (D9, 1172 affected individuals). Phenotypic details of these subcategories are given briefly in Thiselton *et al.* [9].

### Linkage regions

We first limited the ranking to genes in regions with evidence for linkage in the ISHDSF. These regions were obtained from an autosomal genome-wide scan using over 4000 SNPs as part of the Multicenter Genetic Studies of Schizophrenia (PI, Douglas F. Levinson, MD) [10]. Regions were defined as genomic segments with nonparametric linkage (NPL) maximum score of at least 2.0 and telomeric and centromeric boundaries of NPLs of 1.0. The detailed genomic locations were provided in File S1. A bioinformatics search of these regions yielded 3819 human protein-coding genes.

### Prior sources of information

**Figure 1:**
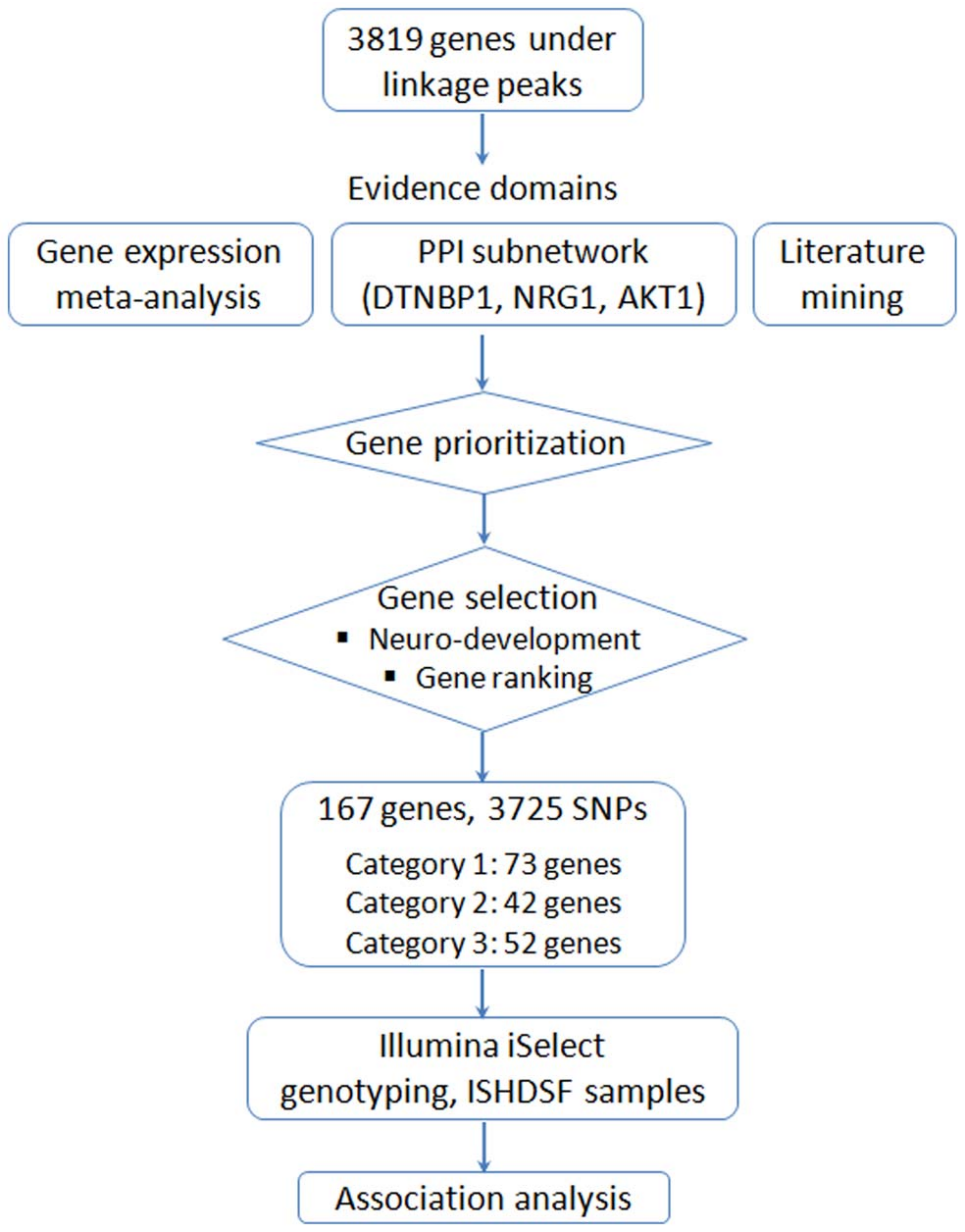
Flowchart of data process, algorithm for gene ranking and selection, custom-based genotyping and association analysis.

For each of the 3819 genes, we obtained a separate p-value pertaining to each of 3 domains: 1) gene expression, 2) protein-protein interaction (PPI) subnetwork, and 3) high-throughput literature search, as illustrated in Figure 1. First, the p-value for fold-change in each gene's expression level was obtained from the Stanley Brain Expression Database (http://www.stanleygenomics.org), which contains meta-analysis results using data from 12 different studies and 988 arrays. A False Discovery Rate (FDR) procedure [11] was applied to the uncorrected p-values and used to generate a corrected ranked p-value. Second, assuming disease genes may be functionally connected, we identified the genes whose proteins interact closely with proteins encoded by three established schizophrenia susceptibility genes (*DTNBP1* [12], [13], *NRG1* [14], [15], and *AKT1* [9]) in the PPI network. A comprehensive human PPI network was generated using human PPI data retrieved from NCBI Entrez Gene (February 2007) which summarizes interactions from multiple sources including HPRD (http://www.hprd.org) [16], BioGrid (http://www.thebiogrid.org) [17], [18], and BIND (http://www.bind.ca) [19]. After removing redundant and problematic interactions, 52,288 unique human PPI pairs remained in the network. The program Pajek (http://vlado.fmf.uni-lj.si/pub/networks/pajek/) [20] was used to determine the minimum number of steps between the proteins encoded by *DTNBP1*, *NRG1*, and *AKT1* and every other human gene in the PPI network. Each of the 3819 genes was assigned a rank based p-value based on the number of steps (lowest to highest). The hypothesis is that a gene closer in the network to a probable susceptibility gene is more likely to harbor susceptibility alleles. Finally, high-throughput literature searching was performed using a Perl script which automatically queried the PubMed database (http://www.ncbi.nlm.nih.gov/pubmed/) for each of the 3819 genes along with 29 schizophrenia-related search terms that we assembled (3819×29 = 110,751 searches). These search terms were divided into several categories: disease states (e.g., “schizophrenia”, “psychosis”), neurotransmitters (e.g., glutamate, dopamine), neuronal features (e.g., “dendrite”, “axon”), brain development, and brain structures (e.g., “cortex”). Genes were ranked according to the number of categories which yielded positive “hits”, and assigned a ranked p-value.

### Ranking and gene selection

For a final ranking of these genes, we summed the −log_10_ of their p-values on each of the three domains (gene expression, PPI network, and literature search). Two subsets of the 3819 genes were selected for tag-based SNP genotyping and association analysis (see Figure 1). The first set was based on the commonly accepted neurodevelopmental hypothesis of schizophrenia (“hypothesis-based”) [7], [21], [22], where all genes with GO terms that included “nervous system development” or “brain development” were selected. The second set was rank-based and included as many top ranked genes as could be included on the custom array based on the remaining unallocated SNPs. In practice, many of the top ranked genes had already been selected by the hypothesis procedure and were in the first set. This led to 125 of the 151 top ranked genes being selected for genotyping with 52 being exclusively highly ranked without being implicated in neurodevelopment. In summary, among the 167 genes we selected for genotyping, 73 were both highly ranked and involved in neurodevelopment (category 1), while 42 and 52 genes were exclusive to neurodevelopment (category 2) or highly ranked (category 3), respectively.

### SNP selection and genotyping

We then identified the genomic region of each candidate gene based on gene annotation information in the UCSC Genome Browser (UCSC hg17/NCBI Build 35, http://genome.ucsc.edu/). For the genomic regions, we attempted to select all haplotype-tagging genic SNPs within each gene using computer program Tagger [23] (r^2^ = 0.8, minor allele frequency (MAF) = 0.1) and the HapMap data (phase 2, http://hapmap.ncbi.nlm.nih.gov/). Genotyping was conducted by Illumina, Inc. using a custom iSelect array, which employs the Infinium assay. In total, genomic DNA for 1128 individuals was submitted for genotyping. Average genotyping completion rate across all SNPs was 99.97%. Of 1128 samples, 21 failed to yield usable genotypes. Genotypes were examined for apparent Mendelian incompatibilities using PEDCHECK v1.1 [24] and removed for entire families where appropriate. After excluding SNPs failing quality control, 3725 SNPs were available for analysis.

### Association analyses

Association analysis for categorical diagnoses of schizophrenia was performed using PDTPHASE (UNPHASED v.2.404), an implementation of the pedigree disequilibrium test (PDT) with extensions to deal with uncertain haplotypes and missing data [25], [26]. The PDT is an extension of the transmission disequilibrium test (TDT) to examine general pedigree structures and is similarly a test of association in the presence of linkage.

### GWAS datasets

The International Schizophrenia Consortium (ISC) samples were collected from eight study sites in Europe and the US [1]. The samples were genotyped using Affymetrix Genome-Wide Human SNP 5.0 and 6.0 arrays. This data was initially analyzed by ISC [1] and was used here for evaluation. A total of 3322 patients with schizophrenia, 3587 normal controls of European ancestry, and a total of 739,995 SNPs were included in our analysis. To account for potential population sub-structure associated with collection sites, the Cochran-Mantel-Haenszel test was used for a single marker association test [1].

We used two GWAS datasets from the Molecular Genetics of Schizophrenia (MGS): The Genetic Association Information Network (GAIN) dataset for schizophrenia and nonGAIN. The GAIN dataset was genotyped using Affymetrix Genome-Wide Human SNP 6.0 array. Our access to this dataset was approved by the GAIN Data Access Committee (DAC request #4532-2) through the NCBI dbGaP. For optimal comparison with the Irish samples (ISHDSF) genotyped in this study, we used only the GAIN samples of European ancestry. We performed quality control (QC) as follows. For individuals, those with a high missing genotype rate (>5%), extreme heterozygosity rate (±3 s.d. from the mean value of the distribution), or problematic gender assignment were excluded. PLINK [27] was used to compute the identify-by-state (IBS) matrix to pinpoint duplicate or cryptic relationships between individuals. We retained the sample with the highest call rate for each pair of samples with an identity-by-descent (IBD) being greater than 0.185. Principle component analysis (PCA) was performed using the smartpca program in EIGENSTRAT [28] to detect population structure and to allow removal of outlier individuals. Eight significant PCs with the Tracy Widom test p-value<0.05 were used as covariates for logistic regression (additive model). For genotyped SNPs, those with a missing genotype rate >5%, MAF <0.05, or departing from Hardy-Weinberg equilibrium (p<1×10^−6^) were removed. The final analytic dataset included 1158 schizophrenia cases and 1377 controls and a total of 654,271 SNPs. The genomic inflation factor (λ), which was defined as the ratio of the median of the empirically observed distribution of the test statistic to the expected median and an indication of the extent of excess false positive rate [29], was 1.04. This value indicates little (if any) inflation.

The MGS - nonGAIN dataset was genotyped in the same laboratory as the MGS -GAIN, but in different phases. Access to this dataset was approved by dbGaP (DAC request #4533-3). Similar QC and PCA processes were peformed as described for GAIN. These processes retained 1068 cases and 1268 controls and 623,059 SNPs for subsequent analyses. Fifteen significant PCs with the Tracy-Widom test p value<0.05 were used as covariates for logistic regression (additive model) using PLINK. The genomic inflation factor (λ) was 1.04.

CATIE (Clinical Antipsychotic Trials of Intervention Effectiveness) is a multi-phase randomized controlled trial of antipsychotic medications involving 1460 persons with schizophrenia. CATIE GWAS included 492,900 SNPs genotyped in a total of 738 cases and 733 group-matched controls using the Affymetrix 500K two-chip genotyping platform plus a custom 164K fill-in chip [30]. Access to this dataset was approved by the National Institute of Mental Health (NIMH) Schizophrenia Genetics Initiative.

### Imputation and meta-analysis

The three GWAS datasets, ISC, GAIN, and nonGAIN, were genotyped on the same Affymetrix platform. To make the data from these GWAS datasets comparable with our custom-design SNPs, we conducted imputation analysis using the HapMap genotyping data for CEU population (release 24) as reference panel. We predicted the genotyping data for a total of 66 SNPs involved in 22 genes using the tool *impute2* [31]. Frequentist association test was then conducted for SNP association using the tool *snptest* [32] by the option “-frequentist 1”, and a missing data likelihood score test for the imputed genotypes by the option “-method score”.

We conducted meta-analysis of candidate SNPs using the imputed data. We performed inverse-variance weighted meta-analysis based on the fixed-effects model using the tool *meta* (http://www.stats.ox.ac.uk/~jsliu/meta.html). This method combines study-specific beta values under the fixed-effects model using the inverse of the corresponding standard errors as weights. Between-study heterogeneity was tested based on I^2^ and Q statistics. SNPs having possible evidence of heterogeneity (*p*_heterogeneity_<0.05) were removed.

### Gene set simulations

In order to determine how often the observed enrichment in p-values would occur, 100,000 simulations were performed where the same number of genes was randomly chosen from the CATIE and GAIN GWAS results. Then, p-values less than 0.05 and 0.005 for genotyped SNPs that mapped to the randomly chosen genes were counted. Due to the great variation in gene size, SNP density per gene, and difference in arrays used in each GWAS, the number of SNPs in each iteration of the simulations could vary. Therefore, we examined whether the observed number of SNPs for the real set of ranked genes was similar to randomly selected sets. The empirical significance for SNP count, minimum p-value, and p-values below a given threshold were calculated both using all simulations and restricted to those simulations where the SNP count was not significantly different from observed. The empirical significance was calculated using the number of simulations greater than or equal to the observed plus one divided by the total number of simulations as per North *et al.* [33].

## Results

Figure 1 summarizes the data process, algorithm for gene ranking and selection, custom-based genotyping and association analysis. We analyzed 3725 SNPs covering 167 prioritized genes whose genotypes were examined in 1107 individuals from the 265 high-density schizophrenia families using a custom Illumina iSelect array. This gene list included 115 genes selected by neurodevelopmental hypothesis and 125 genes selected by gene ranking algorithm – 73 were common between these two selection categories (see Materials and Methods). The minimum p-value among these 3725 tested SNPs was 0.000536 in gene *PRKG1* (SNP rs1904687). This gene was chosen as part of the neurodevelopmental hypothesis since it ranked only 954^th^ of the 3819 genes. Table 1 shows the genes with at least one SNP whose p-value was <0.01 and their test category and rank. Results for all SNPs tested are available in File S1. Although there were three SNPs with p-values less than 0.001 in *PRKG1*, 247 SNPs were tested in this large gene. Therefore, none of the SNPs were significant after gene- or experiment-wide correction for multiple testing (Bonferroni correction, which is a stringent correction). A False Discovery Rate (FDR) analysis of all tests also supported this conclusion, with a minimum FDR based q-value of 0.719.

**Table 1:** Summary of genes with at least one significant (p<0.01) SNP.

Gene	Category	Rank	# SNPs	Min p-value
*PRKG1*	2	954	247	0.000536
*PRKCE*	1	35	229	0.001321
*CNTN4*	2	645	381	0.001474
*EMX1*	2	549	2	0.001949
*INSR*	1	109	51	0.001975
*XPO1*	3	88	2	0.001978
*VAV3*	3	98	76	0.002273
*UTRN*	3	99	56	0.002886
*SH3GL2*	2	538	74	0.003219
*NPAS2*	3	96	55	0.003419
*IL19*	3	149	8	0.003559
*EGFR*	3	8	67	0.004041
*NRP1*	2	291	56	0.004202
*HLA-DRA*	3	144	11	0.004317
*MAL*	2	361	4	0.004592
*CNTN6*	2	862	146	0.005419
*VAV2*	1	132	66	0.006312
*PPP2R2B*	3	151	47	0.007685
*IKBKB*	1	65	4	0.008221
*MGMT*	3	78	52	0.008672
*NOTCH1*	2	439	15	0.009058
*GRIA1*	3	101	67	0.009981

Category 1: genes are both highly ranked and involved in neurodevelopment. Category 2: genes are exclusive to neurodevelopment. Category 3: genes are exclusively highly ranked (see details in Materials and Methods).

A number of genes, including *PRKG1*, *CNTN4*, and *PRKCE*, contained clusters of nominally significant SNPs (p<0.01): 17 SNPs in *PRKG1*, 9 SNPs in *CNTN4*, and 5 SNPs in *PRKCE* (see File S2). Of these three genes, two were from the neurodevelopmental set exclusively and one (*PRKCE*) was from the combined hypothesis and highly rank categories. Gene rank was not correlated with the minimum p-value observed in the tested genes. There was an enrichment of significant p-values in the neurodevelopment group; however, surprisingly, the ranked genes performed worse than randomly selected genes. A summary of the number of genes, SNPs, and p-values by category is provided in Table 2.

**Table 2:** Summary of genes and number of SNPs per test category.

Category	# genes	# SNPs	Highly ranked	Neuro-development	Mean p-value	SNPs with p<0.05		SNPs with p<0.005	
						Obs.	Exp.	Obs.	Exp.
1	73	1271	Yes	Yes	0.521	42	63.6	4	6.4
2	42	1525	No	Yes	0.480	103	76.3	17	7.6
3	52	929	Yes	No	0.488	63	46.5	2	4.6
1+2	115	2796	-	Yes	0.498	145	139.8	21	14.0
1+3	125	2200	Yes	-	0.507	105	110.0	6	11.0
All	167	3725				208	186.3	23	18.6

Category 1: genes are both highly ranked and involved in neurodevelopment. Category 2: genes are exclusive to neurodevelopment. Category 3: genes are exclusively highly ranked (see details in Materials and Methods).

### Comparison with published schizophrenia GWAS results

We compared the current results to two published schizophrenia GWAS datasets, the Clinical Antipsychotic Trials of Intervention Effectiveness (CATIE) GWAS dataset [30] and the GAIN dataset from Molecular Genetics of Schizophrenia (MGS) [2] (see Materials and Methods). Simulated gene selection was used to determine how often the observed enrichment would occur if the whole genome was assessed. Lists of genes were randomly selected 100,000 times using the same numbers of genes selected in the three selection categories (73, 42, and 52 for categories 1, 2, and 3, respectively). Unfiltered simulation showed that the number of SNPs per gene in the current study was significantly higher than for randomly selected genes from either the CATIE or GAIN study. Therefore, the tests for significant enrichment of p-values below 0.05 and 0.005 are biased in the unfiltered simulations. To reduce the bias, random gene sets were ranked based on the total number of SNPs. Different rank filtering thresholds were tested until there was no significant difference in total number of SNPs between the observed and simulated sets. The rank filter thresholds necessary to achieve non-significance were quite different for the CATIE and GAIN studies with the top 500 and 10,000 being used respectively. The filtered simulations showed the ‘neurodevelopment only’ category to be significantly (p = 0.012) and marginally (p = 0.058) enriched for p-values less than 0.005 in the CATIE and MGS-GAIN samples, respectively. Further details of the simulation results are in Table 3.

**Table 3:** Comparison of ISHDSF rank and hypothesis based gene selection results to random gene selection in schizophrenia CATIE and GAIN GWAS datasets.

Category^a^	Simulation		Empirical p-value				
	Method^b^	Observed in ISHDSF	CATIE GWAS		GAIN GWAS		
			100,000^c^	Top 500^d^	100,000^c^	Top 10,000^d^	Top 500^d^
All	SNP count	3741	0.00097	0.194	0.066	0.659	0.998
	min p-value	0.000582	0.618	0.896	0.787	0.879	0.964
	# SNPs with p<0.05	208	**0.0019**	0.208	0.158	0.767	1
	# SNPs with p<0.005	23	**0.0258**	0.234	0.241	0.614	0.924
1	SNP count	1271	0.079	0.655	0.389	0.806	0.892
	min p	0.001975	0.729	0.872	0.859	0.920	0.956
	# SNPs with p<0.05	42	0.586	0.914	0.887	0.976	0.986
	# SNPs with p<0.005	4	0.556	0.836	0.780	0.895	0.934
2	SNP count	1525	0.0022	0.104	0.017	0.121	0.267
	min p	0.000582	0.214	0.349	0.319	0.398	0.445
	# SNPs with p<0.05	103	**0.00042**	**0.032**	**0.017**	0.102	0.232
	# SNPs with p<0.005	17	**0.00294**	**0.012**	**0.018**	0.058	0.142
3	SNP count	929	0.088	0.591	0.358	0.707	0.822
	min p	0.003559	0.797	0.926	0.883	0.932	0.972
	# SNPs with p<0.05	63	**0.049**	0.427	0.264	0.558	0.719
	# SNPs with p<0.005	2	0.701	0.896	0.837	0.914	0.954

a Category 1: genes are both highly ranked and involved in neurodevelopment. Category 2: genes are exclusive to neurodevelopment. Category 3: genes are exclusively highly ranked (see details in text).

b We performed simulations by four methods: 1) based on the count of SNPs, 2) based on the minimum p-value, 3) based on the number of SNPs with p<0.05, and 4) based on the number of SNPs with p<0.005.

c 100,000 simulations (see text).

d To reduce bias, simulations were filtered with top 500 or 10,000 SNPs being used (see Materials and Methods).

### Meta-analysis

In our SNP list, there were 66 SNPs with p<0.01. These SNPs belonged to 22 genes. We examined them in a meta-analysis using three schizophrenia GWAS datasets (ISC, GAIN and nonGAIN). Using the inverse-variance weighted meta-analysis method, we identified 3 SNPs in 3 genes that showed nominal significance (p<0.05) (Table 4). None of them had significant heterogeneity by heterogeneity test. These SNPs are rs2176348 in *PRKCE* (p-value = 0.044), rs552551 in *MGMT* (p-value = 0.044), and rs2616591 in *CNTN4* (p-value = 0.048). Another SNP, rs2043534 in *NPAS2*, had marginal significance (p-value = 0.062). However, none of these SNPs passed Bonferroni multiple testing correction.

**Table 4:** Four SNPs from the meta-analysis of 66 SNPs using GAIN, nonGAIN, and ISC GWAS datasets.

Gene	SNP ID	Chr.	Position (bp)	Allele	Meta-analysis								
					p-value	Beta	s.e.	p_heterogeneity_	I^2^	p_GAIN_	p_nonGAIN_	p_ISC_	IHDS min p
*PRKCE*	rs2176348	2	45798033	A/G	0.044	−0.064	0.032	0.838	0	0.470	0.599	0.057	0.004
*MGMT*	rs552551	10	131271915	C/T	0.044	0.073	0.036	0.29	19.15	0.996	0.797	0.011	0.009
*CNTN4*	rs2616591	3	2614861	C/T	0.048	0.088	0.044	0.481	0	0.347	0.061	NA	0.004
*NPAS2*	rs2043534	2	100847317	C/T	0.062	0.064	0.035	0.635	0	0.258	0.808	0.081	0.003

Chr.: chromosome. GAIN, nonGAIN and ISC are three GWAS datasets for meta-analysis. ISHDSF min p was the smallest p-value in the gene from the IHDS dataset (this study). NA: this SNP was not analyzed in ISC due to missing genotyping data in samples.

We further examined the association signals of these SNPs using the data from the Psychiatric Genomics Consortium (PGC), the largest and most comprehensive cohort dataset for schizophrenia association studies so far [34]. Among the 66 SNPs, 24 were not available in the public release of the PGC dataset (https://pgc.unc.edu/Sharing.php); thus, they could not be imputed due to the lack of access to PGC's raw genotyping data. For the 42 SNPs that had p-values in the PGC dataset, we found that three were nominally significant (p<0.05), including one SNP (rs2616591) in Table 4. Of note, for the four SNPs that were significant or marginally significant in the meta-analysis of our SNPs (Table 4), two were available in the PGC dataset including SNP rs2616591 that had a small p value (1.68×10^−3^).

## Discussion

In this study, we attempted to develop gene ranking strategies based on either evidence from multiple domains (meta-analysis of gene expression, proteins closely interacting with well-studied schizophrenia susceptibility genes, and a systematic literature search) or the neurodevelopmental hypothesis and then applied them to the genes under linkage peaks in the Irish Study of High-Density Schizophrenia Families. For the top ranked genes, we tested their associations with schizophrenia using a custom Illumina iSelect array. The association signals were further evaluated using three GWAS datasets (ISC, GAIN, and nonGAIN). Although none of the SNPs were robustly associated, clusters of significant SNPs were found in several large genes including *PRKG1*, *CNTN4*, and *PRKCE*. These genes were tested not due to rank but as part of the neurodevelopmental hypothesis. This category showed enrichment for significant association signals, and simulations showed this enrichment is unlikely to be due to chance.

There is additional evidence that makes the top results of interest in addition to the reason they were originally tested. CNTN4 (contactin 4) is a neural cell adhesion molecule whose gene has been reported to be associated with autism and developmental delay in multiple studies [35], [36], [37]. Interestingly, another member of the contactin family, *CNTNAP2*, has been found to be associated with both schizophrenia and autism [38]. PRKG1 and PRKCE are known as protein kinase cGMP-dependent, type I and protein kinase C epsilon, respectively. Although they are both protein kinases, they are functionally distinct and activated via different mechanisms. PRKG1 is dependent on cyclic GMP for activation while PRKCE is activated by calcium and the second messenger diacylglycerol. *PRKG1* has previously shown its association with schizophrenia with the 21^st^ most significant SNP in the CATIE GWAS [30]. PRKG1 also interacts with RGS2 and GABRR1, which have shown modest association with schizophrenia symptoms [39] and schizoaffective disorder [40], respectively. Finally, PRKG1 can attenuate beta-catenin expression [41], which is a known downstream target of antipsychotics [42]. PRKCE interacts with several proteins encoded by genes of potential relevance to psychiatric disorders, including the glutamate decarboxylases (GAD1, GAD2), NMDA receptors (GRIN2D, GRIN1), and a metabotropic glutamate receptor (GRM5). PRKCE is also activated by the stimulation of nicotinic receptors [43]. Although these genes were not highly ranked, prior evidence makes them all plausible candidates for schizophrenia. Therefore, each could be chosen using expanded sources of prior information and a refined ranking procedure.

There are several limitations to the current work that could be potentially improved in future application. First, the primary filtering of genes in the genome was done using linkage results from the ISHDSF. Due to the large number of risk variants in schizophrenia, there are likely to be many true associations outside of these regions. The second limitation is the small number of genes and minimum step approach used for the PPI network sets. We used three well-studied genes (*DTNBP1*, *NRG1*, and *AKT1*) in this work. More informative genes including microRNA genes (e.g., *miR-137* [34], [44] and *TCF4* [3], [34]) were recently reported to be associated with schizophrenia and could improve this approach. Larger networks or results from more comprehensive network analyses are probably superior; nevertheless, this work proves the concept using more closely related genes in the PPI network. Third, while our keyword-based literature search seemed to be useful, it might include underpowered studies, negative findings, or studies with methodological flaws or reported false positive results. This is a common problem in literature mining, which could be improved by careful manual check or advanced literature mining technologies like natural language processing (NLP). In our study, gene ranking was performed by the combined evidence from three domains (gene expression meta-analysis, PPI subnetwork, and literature mining). This strategy might help reduce the noisy data from literature mining. Finally, besides the well-supported neurodevelopmental hypothesis, we may test other hypotheses or the candidate genes for samples with refined characterization of phenotypic spectrum. For example, Greenwood et al. [45] recently tested a set of schizophrenia candidate genes in schizophrenia-related endophenotypes, suggesting both converging and independent genetic pathways mediating schizophrenia risk and pathogenesis.

There are several ways to improve or expand the gene selection and prioritization approaches. First, we may develop a more comprehensive data integration approach. This includes the integration of data from multiple domains such as gene expression, copy number variation (CNV), methylation, microRNA, association results, etc. This has been demonstrated in our weight matrix approach for evidence scores [46], as well as other approaches like convergent analysis [47], [48], [49] and microRNA regulatory network analysis [50]. Of note, *TCF4* gene, along with three other genes reported in the PGC meta-analysis (*CACNA1C*, *CSMD1* and *C10orf26*), has predicted *miR-137* target sites [34]. This makes the microRNA-mediated regulatory analysis promising in schizophrenia. In terms of the algorithm, we may apply Bayesian approach, or a comprehensive network and pathway approach, to those multi-domain datasets, since the underlying biological information and regulation is expected to be much related in a complex disease. For example, we recently demonstrated our network approach in schizophrenia [51]. Such approach can be expanded in future by including transcriptional (transcription factors, methylation) and post-transcriptional (microRNA) regulation. Second, with the rapid advances in high throughput technologies, such as Exome chip or next-generation sequencing, we may prioritize the candidate genes that show association signals detected by both common and rare variants and that are involved in disease-related altered genomic regions such as CNVs or structural variants (SVs). This approach benefits from cross-platform and cross-study validation.

In summary, we did not find compelling association evidence for any individual gene selected either by evidence-based gene-ranking or by the rank based on its relevance to the neurodevelopmental hypothesis. However, the neurodevelopmental set of genes showed enrichment for significant associations when examined *en masse*. Finally, several tested genes have additional independent evidence not used in the ranking that make them attractive candidates for further investigation.

## Supporting Information

File S1 This file includes 26 linkage peaks (genomic regions) with nonparametric linkage (NPL) maximum score being at least 2 and telomeric and centromeric boundaries of NPLs of 1.0.
(XLS)

File S2 This file includes details of the 167 genes ranked by three categories and their SNPs with association results in The Irish Study of High Density Schizophrenia Families (ISHDSF) samples.
(XLSX)
